# Oxygenic photosynthesis as a protection mechanism for cyanobacteria against iron-encrustation in environments with high Fe^2+^ concentrations

**DOI:** 10.3389/fmicb.2014.00459

**Published:** 2014-09-02

**Authors:** Danny Ionescu, Bettina Buchmann, Christine Heim, Stefan Häusler, Dirk de Beer, Lubos Polerecky

**Affiliations:** ^1^Microsensor Group, Max-Planck Institute for Marine MicrobiologyBremen, Germany; ^2^Department of Stratified Lakes, Leibniz Institute for Freshwater Ecology and Inland FisheriesStechlin, Germany; ^3^Department of Geobiology, Geoscience Center, Georg-August University of GöttingenGöttingen, Germany; ^4^Department of Earth Sciences – Geochemistry, Faculty of Geosciences, Utrecht UniversityUtrecht, Netherlands

**Keywords:** oxygenic phototrophs, Cyanobacteria, Fe(II), iron-encrustation, banded iron formations, oxygen microgradients, pH microgradients

## Abstract

If O_2_ is available at circumneutral pH, Fe^2+^ is rapidly oxidized to Fe^3+^, which precipitates as FeO(OH). Neutrophilic iron oxidizing bacteria have evolved mechanisms to prevent self-encrustation in iron. Hitherto, no mechanism has been proposed for cyanobacteria from Fe^2+^-rich environments; these produce O_2_ but are seldom found encrusted in iron. We used two sets of illuminated reactors connected to two groundwater aquifers with different Fe^2+^ concentrations (0.9 μM vs. 26 μM) in the Äspö Hard Rock Laboratory (HRL), Sweden. Cyanobacterial biofilms developed in all reactors and were phylogenetically different between the reactors. Unexpectedly, cyanobacteria growing in the Fe^2+^-poor reactors were encrusted in iron, whereas those in the Fe^2+^-rich reactors were not. *In-situ* microsensor measurements showed that O_2_ concentrations and pH near the surface of the cyanobacterial biofilms from the Fe^2+^-rich reactors were much higher than in the overlying water. This was not the case for the biofilms growing at low Fe^2+^ concentrations. Measurements with enrichment cultures showed that cyanobacteria from the Fe^2+^-rich environment increased their photosynthesis with increasing Fe^2+^ concentrations, whereas those from the low Fe^2+^ environment were inhibited at Fe^2+^ > 5 μM. Modeling based on *in-situ* O_2_ and pH profiles showed that cyanobacteria from the Fe^2+^-rich reactor were not exposed to significant Fe^2+^ concentrations. We propose that, due to limited mass transfer, high photosynthetic activity in Fe^2+^-rich environments forms a protective zone where Fe^2+^ precipitates abiotically at a non-lethal distance from the cyanobacteria. This mechanism sheds new light on the possible role of cyanobacteria in precipitation of banded iron formations.

## Introduction

Abiotic oxidation of Fe^2+^ to Fe^3+^ and subsequent precipitation of FeO(OH) is a function of pH and O_2_ concentration, and is relatively rapid when O_2_ is available at circumneutral pH (Khalil et al., 2011). To prevent iron self-encrustation, iron oxidizing bacteria developed a variety of protection mechanisms, including formation of organic matter stalks (Chan et al., 2009; Suzuki et al., 2011) or sheaths (Van Veen et al., 1978) that provide a template for FeO(OH) nucleation. For some phototrophic iron oxidizers, a low-pH microenvironment generated by the cell's proton pumps has been suggested as a mechanism preventing iron self-encrustation (Hegler et al., 2010). Additional adaptations include a hydrophilic cell membrane with a near neutral surface charge to prevent the adhesion of Fe^2+^/Fe^3+^ (Saini and Chan, 2013).

Oxygenic phototrophs are often found in microbial mats in Fe^2+^-rich environments (Pierson et al., 1999; Brown et al., 2005, 2010; Wieland et al., 2005). It is known that when the photosynthetically active cells are densely packed in a volume where transport is limited by diffusion (e.g., in photosynthetic biofilms or mats), their activity leads to a locally increased pH and O_2_ concentration (Pierson et al., 1999; Wieland et al., 2005), which should favor locally higher rates of Fe^2+^ oxidation and precipitation. Yet, oxygenic phototrophs in Fe^2+^-rich environments have seldom been found encrusted in precipitated iron (Pierson and Parenteau, 2000). A defense mechanism against iron self-encrustation that would enable oxygenic phototrophs to thrive in Fe^2+^-rich environments has hitherto not been suggested. Although cyanobacteria that accumulate iron precipitates intracellularly were described from Yellowstone National Park (Brown et al., 2010), this phenomenon has not been found elsewhere.

In the Fe^2+^-rich environment of the Äspö Hard Rock Laboratory (HRL), Sweden, which is a man-made research tunnel, a series of flow-through reactors were set up in 2006 as part of a study on iron oxidizing bacteria (Ionescu et al., 2014a). Part of the reactors were connected to two aquifers that emerge from the tunnel wall at the top and bottom of the tunnel and differ markedly with respect to concentrations of dissolved Fe^2+^ (top: ~26 μM, bottom: ~0.9 μM). Half of the reactors were illuminated and the other half were not. After four years of undisturbed incubation, we found that inside the illuminated reactors cyanobacterial biofilms developed. Interestingly, cyanobacteria in the Fe^2+^-poor reactors were mostly encrusted in iron precipitates, whereas those from the Fe^+2^-rich reactors were not.

In this study we aimed to understand this counter-intuitive observation. Past studies showed that only some morphotypes of cyanobacteria are found encrusted in iron (Pierson and Parenteau, 2000; Parenteau and Cady, 2010) while others are not; however a mechanism to account for these differences was not suggested. Pierson et al. (1999) showed that cyanobacterial mats from iron rich environments were characterized by high photosynthetic O_2_ production, which was similar to our observation in the cyanobacterial biofilms growing in the Fe^2+^-rich reactors. Hence, we hypothesized that it is the high rate of oxygenic photosynthesis that allows cyanobacterial proliferation in the Fe^2+^-rich environment of the Äspö HRL. Specifically, due to mass transfer limitations, cyanobacterial photosynthesis creates a microenvironment with elevated O_2_ concentrations and pH that causes high rates of Fe^2+^ oxidation and precipitation at a non-lethal distance from the cells and thus prevents their iron self-encrustation.

## Materials and methods

### Reactors

The Äspö HRL is a man-made research tunnel (length 3.6 km) operated since 1995 by the Swedish Nuclear Waste Management Facility (SKB) in the south-east of Sweden. The tunnel intersects several groundwater aquifers, each characterized by specific water chemistry (Laaksoharju et al., 1999; Ionescu et al., 2014a). In 2006 two sets of two illuminated reactors were connected directly to the aquifers at sites TASF (depth 460 m) and TASA-1327B (depth 185 m) using valves KF0069A01 and HA1327B, respectively. Only chemically inert materials such as polytetrafluoroethylene (PTFE, Teflon^®^), PTFE-fiber glass, fluorinatedethylene propylene (FEP) and special PTFE-foam were used as construction materials to avoid biological contamination from the surrounding environment and chemical contamination from glass- and plastic-ware. The reactors and connection tubing were sterilized with ethanol (70%, overnight) before underground installation. In each set, reactor 1 (R1) had an air headspace and gas exchange was allowed through a 0.2 μm membrane filter. Reactor 2 (R2) did not have an air headspace, which was achieved by elevating the outflow pipe above the height of the reactor. Both reactors were illuminated using two fluorescent lamps (WL11, Brennenstuhl, Tübingen, Germany; Figure S1). Light penetrated inside the reactors through an FEP-window in the center of the lid. Irradiance behind the window was 60 μmol photons m^−2^ s^−1^, as determined by a quantum irradiance sensor (LI-190 Quantum) connected to a light meter (LI-250, both from LI-COR Biosciences).

### DNA extraction and sequencing

To characterize the composition of the cyanobacterial communities developed in the reactors, DNA was extracted as previously described (Ionescu et al., 2012). Pyrosequencing was done by MrDNA laboratories (Shallowater, TX) using general bacterial primers 27F and 519R (Lane, 1991). Sequences were analyzed using the SILVA NGS pipeline (Ionescu et al., 2012) and the SILVA 111 database (Quast et al., 2012).

### Culturing

Green cyanobacterial biomass that developed in the aerated Fe^2+^-poor (TASF-R1) and aerated/non-aerated Fe^2+^-rich (1327-R1/R2) reactors was physically separated from the rusty-orange biomass of iron oxidizing bacteria and transferred on site to sterile BG-11 medium (Rippka et al., 1979). Upon arrival to the laboratory the samples were transferred to both solid and liquid BG-11 medium and grown in constant light (irradiance 60–70 μmol photons m^−2^ s^−1^) at 15°C to provide light and temperature conditions close to those in their natural habitat. To allow for later microsensor measurements (see below) without the loss of added iron, cultures were allowed to grow on GF/F filters (Whatmann, diameter 47 mm) that were soaked in Fe^2+^-free media and placed on top of pre-grown BG-11 (Fe^2+^-free) agar plates. Filters were used once the green cyanobacterial biomass was visible.

### Microsensor measurements

Dissolved O_2_ concentration was measured with a fast-responding Clark-type microelectrode (tip diameter ~30 μm). pH was measured using shielded liquid ion exchange glass microelectrodes. Both microsensors were constructed and calibrated as previously described (Revsbech, 1989; de Beer and Stambler, 2000). *In-situ* measurements were conducted either directly in the reactors or, if not possible, directly next to them, placing the biofilms in water from the respective reactor. Illumination during all experiments was provided by a Schott KL1500 lamp (Carl Zeiss AG, Göttingen, Germany), with the incident irradiance adjusted to match the value inside the reactors (60 μmol photons m^−2^ s^−1^).

For laboratory measurements 0.5 cm filter stripes with a well-developed cyanobacterial biomass were placed in an equally wide flow-through chamber (volume ~5 mL) connected to a media reservoir using a peristaltic pump (Figure S2). The medium was continuously purged with N_2_ gas to maintain anoxic conditions. During measurements, Fe^2+^ was added to the media reservoir while subsamples for iron determination were periodically taken from the chamber to assure the required concentration (1, 5, 10, 30, 50 μM) is maintained. Iron was added from an acidic Fe(II)Ammonium Sulfate stock solution resulting in a slight acidification of the medium (<0.2 pH units). Used medium was not recycled.

A similar iron addition experiment was performed also *in-situ* using a biofilm collected from the aerated Fe^2+^-poor reactor (TASF-R1). The sample was placed in a 1 L beaker and covered with water from the same reactor. The water was purged with N_2_ and iron was added to a final concentration of 25 μM.

In all experiments Fe^2+^ was measured colorimetrically using ferrozine as previously described (Riemer et al., 2004).

### NanoSIMS measurements

To test whether iron oxides are being precipitated inside cyanobacterial filaments in a similar way as described by Brown et al. (2010), ^57^Fe^2+^ was added to a culture from the non-aerated Fe^2+^-rich reactor at a concentration of 50 μM. Samples were transferred to polycarbonate filters and cyanobacterial filaments were identified using auto-fluorescence. Areas that contained the filaments were marked by a laser dissection microscope (LMD7000, Leica), which allowed their later localization and identification in the nanoSIMS instrument using the built-in CCD camera. NanoSIMS analysis was performed with the nanoSIMS 50 L instrument (Cameca) available at the MPI Bremen. Before each analysis the sample was pre-sputtered with a Cs^+^ primary ion beam (16 keV, 1.1–3.5 pA) focused on a spot of ~120 nm diameter for 60–90 s. Subsequently, the same beam was scanned over the sample in a 256 × 256 pixel raster with a counting time of 1 ms per pixel, and the following ions were detected: ^12^C^−^, ^12^C^14^N^−^, ^32^S^−^, ^56^Fe^16^O^−^, and ^57^Fe^16^O^−^. The mass resolution power during all measurements was >8000. For each region of interest 30–100 planes were acquired at a raster size of 15 × 15 or 30 × 30 μm. Data analysis was done with the Look@NanoSIMS software (Polerecky et al., 2012). As a control of the method, the same ^57^Fe^2+^-addition experiment and nanoSIMS analysis were performed using samples from mats of iron oxidizing bacteria.

### Fe^2+^ profile modeling

Depth profiles of Fe^2+^ concentrations around the biofilm surface could not be measured and were therefore modeled numerically. First, a depth profile of Fe^2+^ oxidation rates was calculated according to Morgan and Lahav (2007) based on the profile of O_2_ concentrations and pH measured *in-situ*. Subsequently, these rates were used to calculate the evolution of the Fe^2+^ concentration profile, starting from a flat profile (i.e., Fe^2+^ concentrations in the entire modeled domain equal to that measured in the reactor water). Specifically, during each time-step, the net decrease in the Fe^2+^ concentration at a given depth was the result of the local Fe^2+^ oxidation rate combined with the transport by diffusion determined from the Fe^2+^ concentrations above and below. Fe^2+^ concentrations at distances greater than the thickness of the diffusive boundary layer (DBL) were kept constant and equal to the initial concentrations during the entire calculation. DBL thickness was derived from the measured O_2_ profile. Calculation proceeded in time-steps of 1 × 10^−3^ s until a steady state was reached. Steady state was defined when the changes in Fe^2+^ concentration across the entire profile fell below 10^−6^ μM. Equilibrium constants required for the iron speciation were corrected for the ionic strength and temperature of the specific feeding aquifer using the Davies (Davies, 1962) and Van't Hoff (Atkins, 1978) equations, respectively. Ionic strength of the different waters was calculated using Visual MINTEQ (Ver. 3.0). Iron diffusion coefficient was set to 5.82 × 10^−6^ cm^2^ s^−1^ (Yuan-Hui and Gregory, 1974). As no pH profile was available from the aerated Fe^2+^-rich reactor, modeling was done only for biofilms from the non-aerated Fe^2+^-rich and aerated Fe^2+^-poor reactors.

## Results

### Water chemistry

Water in the top aquifer is mainly influenced by the Baltic Sea and recent meteoric water and has a retention time in the bedrock of several weeks (Laaksoharju et al., 1999). It is rich in dissolved Fe^2+^ (around 26 μM) and has pH of 7.4 and O_2_ concentrations of about 2 μM. In contrast, water in the bottom aquifer is a mixture of glacial, ancient marine and brine water and was dated to the Boreal age (SICADA, SKB Database). It has markedly lower Fe^2+^ concentrations (0.9 μM), higher pH (8), and O_2_ concentrations of about 4 μM. Other notable differences include dissolved inorganic carbon (DIC), total alkalinity (Alk), and total dissolved salt (TDS) concentrations, which are about 10- and 100-fold lower and 5-fold higher in the bottom aquifer, respectively (Table 1).

**Table 1:** **Physico-chemical characteristics of the different aquifers connected to the reactors**.

**Site**	**Sampled water**	**pH**	**T °C**	**Fe^2+^ μM**	**O_2_ μM**	**Alk μM**	**DIC μM C**	**DOC μM C**	**TDS μM C**	**H_2_S μM**	**NH_4_ μM**	**NO_3_ μM**
TASF (Fe^2+^-poor)	Aquifer	7.98	12	0.89	3.7	110	133	183	23.6	0.53	0.61	1.05
	R1^#^	7.94	12	0.89	32.5	110	133	167	23.5	0.53	0.67	1.03
TASA (1327B Fe^2+^-rich)	Aquifer	7.41	15	25.8	2.2	3277	1691	492	5.1	1.88	215	0.12
	R1^#^	7.40	15	27.1	7.2	3277	1933	617	5.1	1.17	217	1.15
	R2^#^	7.39	15	26.4	3.9	3245	1825	558	4.9	0.88	212	1.48

# R1 and R2 are aerated and non-aerated flow reactors, respectively.

### *In-situ* biomass

**Figure 1:**
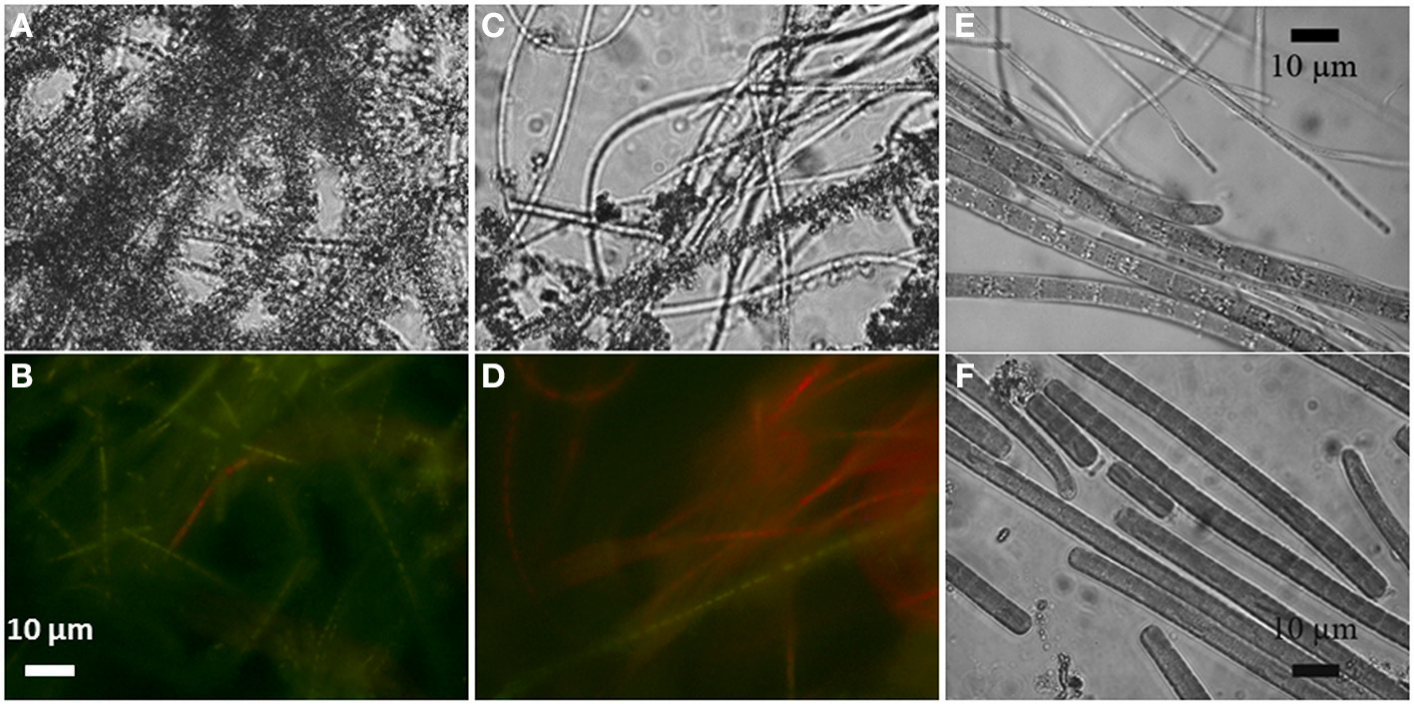
**Light (A) and autofluorescence (B) microscopic images of iron-encrusted cyanobacterial filaments from the aerated Fe^2+^-poor reactor**. Upon treatment with 0.3 M oxalic acid most of the Fe crystals dissolved **(C)** and the natural red autofluorescence induced by green light resumed **(D)**. Filaments from the aerated **(E)** and non-aerated **(F)** Fe^2+^-rich reactors were not found encrusted.

Filamentous cyanobacteria in the aerated reactors connected to the Fe^2+^-poor aquifer formed veil-like biofilms (2–3 cm long, 2–3 mm thick) that slowly but continuously moved due to the slow water flow in the reactor. Their pale-green color coincided with a low chlorophyll *a* (Chl *a*) concentration (0.2 μg Chl *a* mg^−1^ wet weight) and thus presumably low cyanobacterial biomass. Microscopic observations of biofilm subsamples revealed clear iron encrustation and largely diminished auto-fluorescence of the filaments (Figures 1A,B). Upon addition of 0.3 M oxalic acid, most of the Fe-oxide crystals dissolved and the red auto-fluorescence induced by green light, which is typical for cyanobacteria due to their Chl *a* and phycocyanin content, significantly increased (Figures 1C,D). Due to an extremely low biomass, biofilms from the non-aerated reactor from the Fe^2+^-poor site were not studied.

Cyanobacterial biofilms in the reactors connected to the Fe^2+^-rich aquifer had about 10-fold larger biomass (3.2 μg Chl *a* mg^−1^ wet weight), consistent with their dark-green appearance. Biofilms in the aerated reactor were 0.5–1 mm thick, floated atop of black decaying material (2–3 cm thick) and were in direct contact with air in the reactor (but still moist). In contrast, cyanobacterial biofilms in the non-aerated reactor were submersed, about 0.3 mm thick, and covered thick mats of iron oxidizing bacteria, the latter identified based on their rusty-orange appearance and an in-depth community analysis performed earlier (Ionescu et al., 2014a). Importantly, no signs of iron encrustation were observed for the cyanobacteria from these two reactors (Figures 1E,F).

### Cyanobacterial community

Phylogenetic comparison based on the gene for the 16S rRNA revealed that the cyanobacterial communities significantly differed between the reactors from the Fe^2+^-rich and Fe^2+^-poor sites (Figure S3). Communities from the aerated and non-aerated reactors from the Fe^2+^-rich site were more diverse, clustering with sequences of *Geitlerinema, Pseudanabaena*, and several different clusters of *Leptolyngbia*. Many sequences from these reactors were shared. In contrast, cyanobacterial sequences from the aerated reactor from the Fe^2+^-poor site were unique, forming a cluster within the clade of *Leptolyngbia* (Figure S3). No sequences from any of the sampled reactors were closely related with the known ferro-philic cyanobacteria *Chroogloeocystis siderophila.* Sequences of cyanobacterial enrichment cultures from the respective reactors clustered in close proximity to those obtained from *in-situ* samples. One of these sequences was closely related to *Oscillatoria* JSC-1, a cyanobacterial strain known to precipitate iron internally (Brown et al., 2010).

Clustering of the sequences at 98% similarity cutoff using the CDHIT-EST software(Huang et al., 2010; Fu et al., 2012) supports the phylogenetic tree and shows that no overlap is found between sequences from the Fe^2+^-poor and the Fe^2+^-rich reactors. In contrast, sequences from the Fe^2+^-rich aerated and non-aerated reactors form individual as well as shared clusters (data not shown).

### O_2_ and pH microsensor measurements

**Figure 2:**
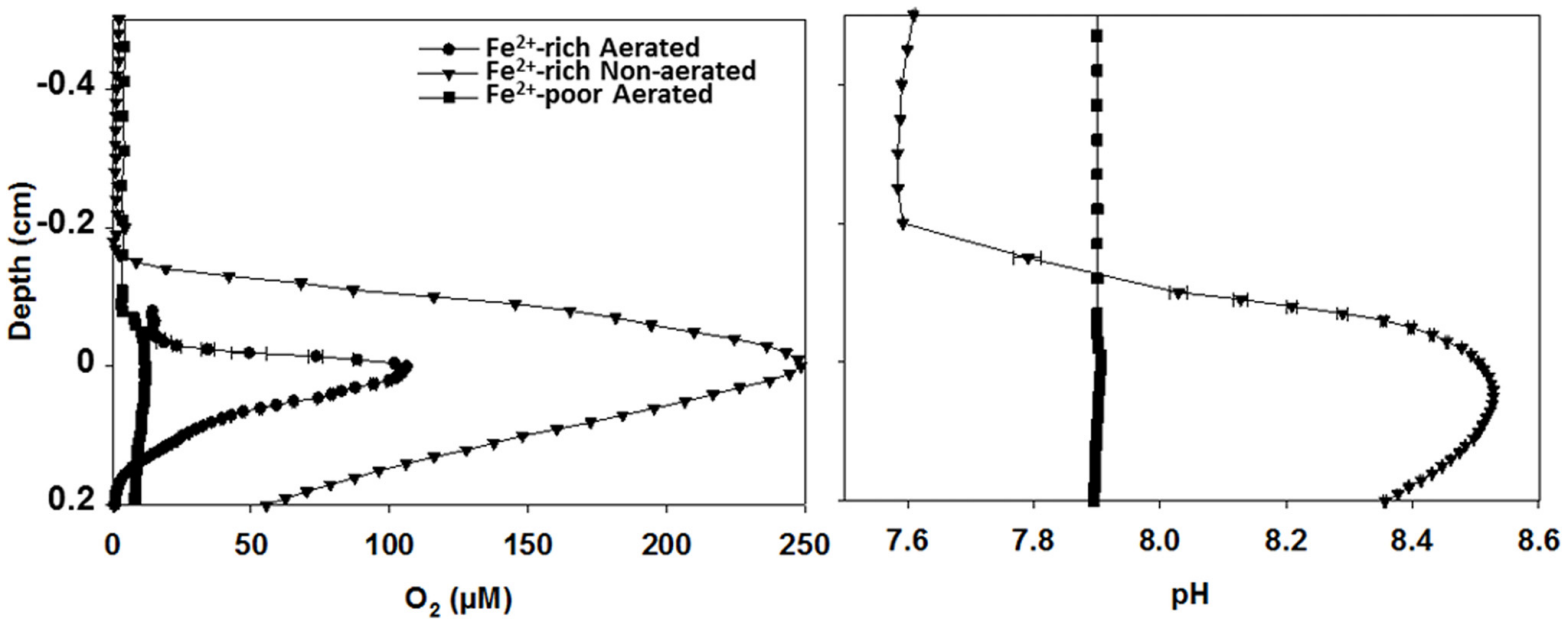
***In-situ* O_2_ and pH microprofiles in cyanobacterial biofilms from aerated and non-aerated Fe^2+^-rich and aerated Fe^2+^-poor reactors**. All profiles were measured under similar irradiance as that used during long-term incubations of the reactors. Measurements in the biofilm from the aerated Fe^2+^-poor reactor were conducted outside of the reactor using the natural water purged with N_2_ gas to maintain anoxic conditions.

*In-situ* microsensor measurements revealed high volumetric rates of oxygenic photosynthesis in the biofilms forming in the Fe^2+^-rich reactors. This was demonstrated by the steep gradients in O_2_ and pH around the biofilm-water interface (Figure 2) as well as by direct rate measurements using the light-dark-shift method of Revsbech and Jorgensen (1981) (Figure S4). In contrast, O_2_ and pH profiles had only minute peaks in the biofilms in the Fe^2+^-poor reactor (Figure 2), indicating very low rates of photosynthesis. Based on the measured O_2_ profiles, the estimated net areal rates of photosynthesis were 21–23 and 1.5–5 μmol m^−2^ s^−1^ in the biofilms from the Fe^2+^-rich and Fe^2+^-poor reactors, respectively. This pattern was consistent with the approximately 10-fold difference in the cyanobacterial biomass (see above).

We used freshly collected subsamples of the biofilms from the Fe^2+^-poor reactor to test the response of their photosynthesis to the addition of Fe^2+^ at a concentration comparable to that found in the Fe^2+^-rich reactors. We found that the photosynthetic O_2_ production started to decrease within minutes after the increase of the Fe^2+^ concentration to 25 μM and was close to zero after about an hour (Figure S5).

**Figure 3:**
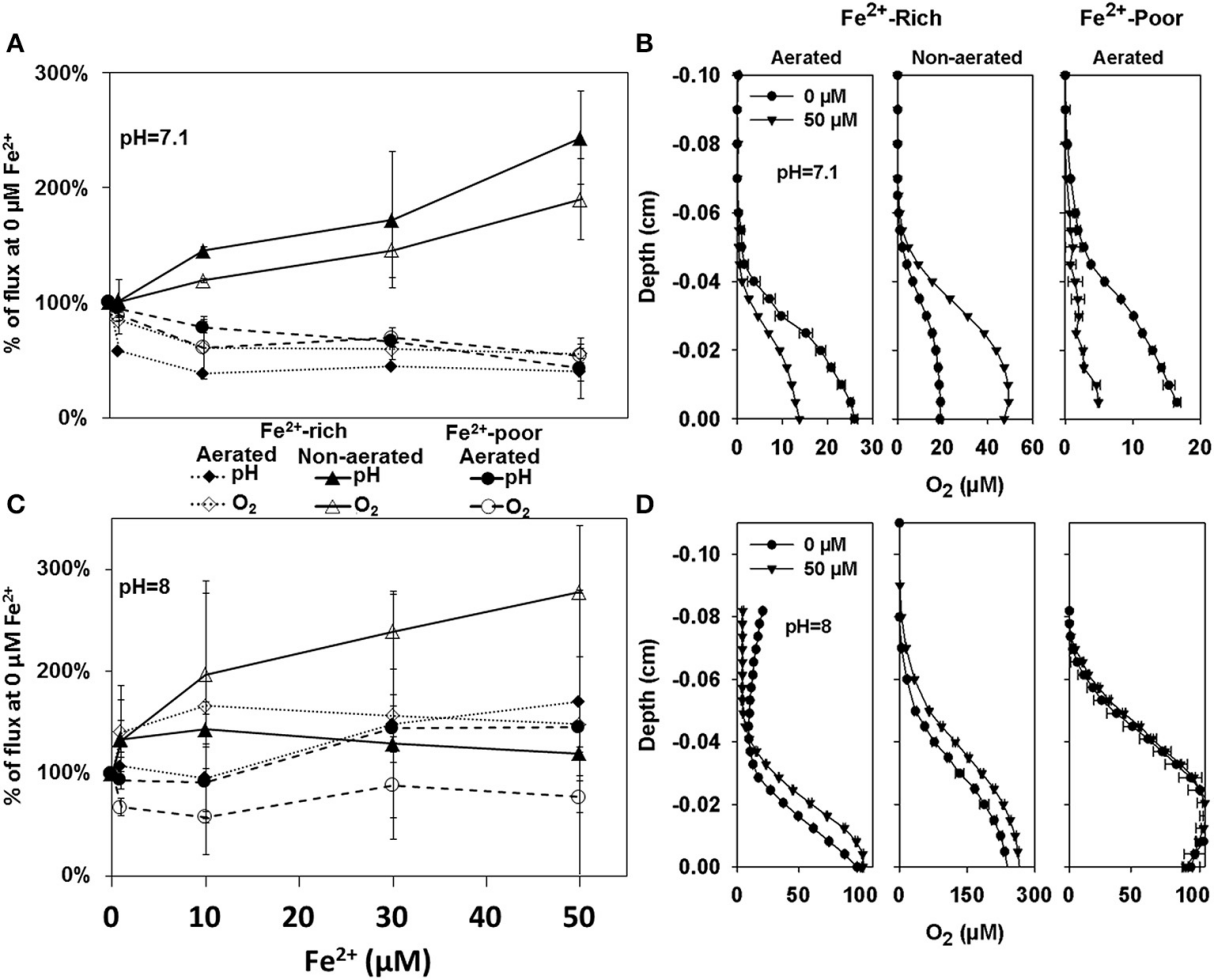
**Percent change in O_2_ fluxes and pH gradients calculated from microprofiles measured in cyanobacterial biofilms grown from enrichment cultures from the Fe^2+^-rich and Fe^2+^-poor reactors at pH 7 (A) and pH 8 (C)**. The shown fluxes are averaged measurements in 3 different biofilms with 3–4 steady state profiles each. Example of profiles from which these fluxes were calculated are shown in **(B,D)**, respectively. Depth zero corresponds to the biofilm surface.

In addition to *in-situ* measurements, we conducted laboratory microsensor measurements to characterize the response of photosynthesis to increased Fe^2+^ concentrations in biofilms prepared from the cyanobacterial enrichment cultures obtained from the two reactor types. When the bulk pH was 7.1, the addition of up to 50 μM Fe^2+^ stimulated net O_2_ production in the enrichment cultures from the non-aerated Fe^2+^-rich reactor (Figure 3A — open triangles; Spearman correlation coefficient ρ = 0.952, *p* = 2 × 10^−7^), suggesting stimulation of photosynthetic activity by Fe^2+^. This was consistent with the observed increase in pH gradients at the biofilm-medium interface (Figure 3A, filled triangles; ρ = 0.854, *p* = 2 × 10^−7^). In contrast, increasing Fe^2+^ lead to a decrease in both O_2_ fluxes and pH gradients in biofilms prepared from the enrichment cultures from the aerated Fe^2+^-rich (O_2_: ρ = −0.686, *p* = 7.45 × 10^−4^; pH: ρ = −0.741, *p* = 0.011) and Fe^2+^-poor reactor (O_2_: ρ = −0.865, *p* = 2 × 10^−7^; pH: ρ = −0.986, *p* = 2 × 10^−7^), with the largest part of this decrease occurring for Fe^2+^ concentrations between 1 and 10 μM (Figures 3A,B). Analysis of covariance (ANCOVA) revealed that the slope of the regression line for the culture enriched from the non-aerated Fe^2+^-rich reactor was significantly greater than that for the enrichment cultures from the aerated Fe^2+^-rich and aerated Fe^2+^-poor reactors (*p* = 2 × 10^−9^), whereas the latter two were not significantly different (*p* = 0.97).

When the same experiments were performed at bulk medium pH of 8, net O_2_ production was again significantly stimulated by Fe^2+^ in the biofilm cultures from the non-aerated Fe^2+^ reactor (ρ = 0.716; *p* = 0.015), while no significant inhibition was observed in the biofilms from the aerated Fe^2+^-rich (ρ = 0.543; *p* = 0.097) and aerated Fe^2+^-poor (ρ = −0.123; *p* = 0.707) reactors (Figures 3C,D). However, due to the high variability in the results, the slopes of the regression lines for the three biofilm types were not significantly different (ANCOVA, *p* = 0.079). Also, there was no significant difference in the slopes determined at pH 7.1 and 8 in the biofilm cultures from the Fe^2+^-rich reactors (ANCOVA, aerated *p* = 0.20; non-aerated *p* = 0.26).

Importantly, no encrustation in iron minerals was observed in any (pH 7.1 or pH 8.0) of these experiments.

### NanoSIMS measurements

No iron precipitates could be detected in or nearby the cyanobacterial filaments from the non-aerated Fe^2+^-rich reactor which were incubated with ^57^Fe^2+^ (Figures S6A,B). This is in contrast to control experiments conducted with samples from mats of iron oxidizing bacteria where some cells were clearly enriched in ^57^Fe precipitates as compared to the surrounding (Figures S6C,D; see also Ionescu et al., 2014b).

### Fe^2+^ profile modeling

**Figure 4:**
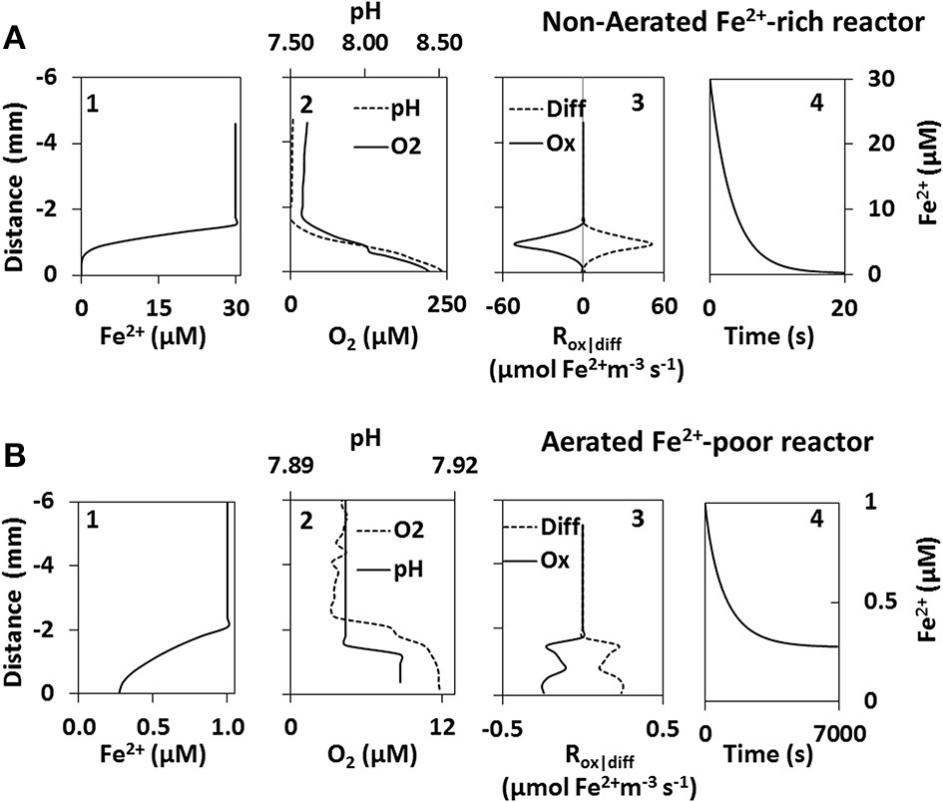
**Fe^2+^ model results of the non-aerated Fe^2+^-rich (A) and aerated Fe^2+^-poor (B) reactors**. Steady state Fe^2+^ profiles above cyanobacteria biofilms (graph 1) were calculated using microprofiles of O_2_ and pH measured *in-situ* (graph 2). Steady state was achieved once Fe^2+^ consumption by abiotic oxidation (Ox) was equal to Fe^2+^ supplied by diffusion (Diff) (graph 3). The change in Fe^2+^ concentration at the biofilm surface is shown as a function of time (graph 4).

Modeling in the non-aerated Fe^2+^-rich reactor revealed that the steady state Fe^2+^ concentration profile was reached in ~10 min. Fe^2+^ concentration at the biofilm surface decreased from the initial value of 30 μM to below 0.001 μM in about 20 s, and in the steady state the Fe^2+^ concentration was below 0.001 μM already at a distance of ~400 μm from the biofilm surface (Figure 4A). In contrast, in the aerated Fe^2+^-poor reactor the steady state was reached after a much longer time (~2 h) and the concentration at the biofilm surface did not decrease below 0.3 μM (Figure 4B).

## Discussion

The role of cyanobacterial oxygenic photosynthesis has been extensively discussed in relation to Fe(III) mineral formation during early Earth (Cloud, 1973; Lewy, 2012). However, the effects of O_2_ production in the presence of high ferrous iron concentrations have been only seldom discussed on the scale relevant for the microenvironment of the cyanobacteria (~100 μm or less) (Pierson and Parenteau, 2000). Our finding of iron-encrusted cyanobacteria at low Fe^2+^ concentrations as compared to naked filaments at high Fe^2+^ concentrations has raised three intriguing questions: (1) Why do we see iron-encrusted filaments specifically in the environment with the lower Fe^2+^? (2) How do oxygenic phototrophs, who by their means of existence form environmental conditions favoring Fe precipitation, avoid encrustation? (3) How do these organisms avoid Fe^2+^ toxicity?

Cyanobacteria are regarded as oxygenic phototrophs, yet a large number of species possess the ability to switch to anoxygenic phototrophy in the presence of sulfide (Cohen et al., 1986; Oren et al., 2005). Therefore, one possible way for photosynthetic cyanobacteria to avoid iron encrustation in Fe^2+^-rich environments could be by performing anoxygenic photosynthesis utilizing H_2_S (detected in the reactors) as the electron donor, which does not lead to O_2_ production. Alternatively, it was suggested that cyanobacteria could also use Fe^2+^ as an electron donor in the process of anoxygenic photosynthesis (Olson, 2006). Such cyanobacteria have, however, not been described yet. If such a mechanism exists, it is not clear where would the oxidized Fe be deposited, as the analogous sulfide oxidizing phototrophs can deposit elemental sulfur both internally (Arunasri et al., 2005) and externally (Ventura et al., 2000).

With this background knowledge, we initially hypothesized that the cyanobacteria in the Fe^2+^-rich reactors performed anoxygenic photosynthesis. This hypothesis was rejected by our *in-situ* microsensor measurements, which clearly showed that they performed oxygenic photosynthesis. This was further supported by our failed attempts to grow the cultured cyanobacteria in the presence of DCMU (3-(3,4-dichlorophenyl)-1,1-dimethylurea), an inhibitor of oxygenic photosynthesis, using either sulfide or iron as the electron donor, questioning the ability of these cyanobacteria to perform anoxygenic photosynthesis.

Recently it was shown that some filamentous cyanobacteria from iron-rich environments accumulate iron-oxides internally (Brown et al., 2010). These cyanobacteria are not full with iron particles as one would expect from an organism that is continuously exposed to high Fe^2+^ concentrations. Therefore, it may be that the creation of iron inclusions is an auxiliary mechanism only. Our NanoSIMS measurements did not detect any accumulation of iron oxides inside the cyanobacteria cells from the Äspö HRL (Figure S6). Such an accumulation would have accounted for the lack of external iron precipitates and may have provided a detoxification mechanism.

The above results call for a different mechanism to prevent encrustation in iron while actively producing O_2_. We suggest that, counter-intuitively, it is the photosynthetic activity itself, in combination with mass transfer limitation, that protects cyanobacteria against iron encrustation.

Our data show that each of the three cyanobacterial communities sampled is adapted to the iron concentrations it lives in. Oxygen production by the cyanobacteria enriched from the Fe^2+^-poor reactor is inhibited by more than 1 μM Fe^2+^, yet they never encounter higher concentrations in their natural habitat. To confirm that this is true also for the original biofilm community from the Fe^2+^-poor reactor and not only a trait of the enriched cyanobacteria, we exposed freshly collected biofilms to iron concentrations present in the Fe^2+^-rich reactors. As expected, photosynthesis was inhibited within minutes of the exposure. Intriguingly, the observed decline in oxygen production was coupled with a transient increase in pH at the biofilm surface (Figure S5) for which we presently do not have an explanation.

The cyanobacteria from the aerated Fe^2+^-rich reactor exhibit as well a reduced O_2_ production when exposed to more than 1 μM Fe^2+^ (at pH 7.1). Though they grow in a reactor connected to an aquifer with high ferrous iron concentration, the cyanobacterial biofilms from the aerated Fe^2+^-rich reactors are found only floating at the reactor water surface atop mats of Fe^2+^ oxidizers, where they are not in direct contact with the aquifer water and therefore also with high Fe^2+^ concentrations.

Notably, the inhibitory effects of iron observed in cultures from the aerated Fe^2+^-rich and Fe^2+^-poor reactors at pH 7.1 were not present when the experiment was conducted at pH 8. At this pH Fe is expected to precipitate faster and thus the cells are not exposed to high concentrations at any time. Nevertheless, the cyanobacteria from the Fe^2+^-poor reactor still get encrusted in iron at a pH of 7.9. This phenomenon is explained by the results of our model as discussed later on.

In contrast to the biofilms from the aerated Fe^2+^-rich and Fe^2+^-poor reactors, the submerged cyanobacterial biofilms from the non-aerated Fe^2+^-rich reactor increase their O_2_ production in response to increasing iron concentration. Nevertheless, despite their tolerance to elevated Fe^2+^ concentrations, these are not required for growth in culture as also confirmed by their continuous activity at all tested Fe^2+^ concentrations.

Modeling of Fe^2+^ concentrations in the non-aerated Fe^2+^-rich reactor suggests that all Fe^2+^ is precipitated at a distance of at least 400 μm from the biofilm surface. Thus, at steady state the cyanobacteria forming the biofilm are not exposed to high Fe^2+^ in their microenvironment. The precipitated Fe(oxy)hydroxides are probably dispersed away by the water movement. Although true steady state conditions would be reached rather slowly (in ~10 min), our simulation suggests that already after about 20 s the biofilm is exposed to little or no Fe^2+^ at all. This suggests the necessity for these cyanobacteria to be able to tolerate high Fe^2+^ concentrations for at least a short period of time (seconds to minutes).

The cyanobacteria in the aerated Fe^2+^-poor reactor are not able to precipitate the Fe^2+^ away from their surface. Even if a theoretical steady-state were reached, which would take about 2 h, Fe^2+^ concentrations inside the biofilm would still be about 0.3 μM. Given the low biomass and the continuous movement of the biofilm, such a steady-state condition can, however, never be reached. Thus, given the already high pH in the water column of this reactor, whatever O_2_ these cyanobacteria produce reacts immediately with the available Fe^2+^ and precipitates on the filaments themselves. Furthermore, Fe^2+^ is known to bind to charged moieties of extracellular polymeric substances (Fortin and Langley, 2005). Therefore, the continuous movement of the biofilms coupled with the low O_2_ production will additionally lead to the adherence of Fe to the filaments. Though these cyanobacteria are able to achieve high biomass in Fe^2+^-poor culture media, an increase in Fe^2+^ above 1 μM inhibits their growth. Therefore these organisms are confined to environments with low Fe^2+^.

High photosynthesis rates appear to be a common trait of benthic cyanobacterial communities described from environments with high Fe^2+^ concentrations. Descriptions that include O_2_ and pH data measured in such systems are, however, rare (Pierson et al., 1999; Wieland et al., 2005). We applied our model on data available for cyanobacterial mats from Chocolate Pots (Yellowstone National Park, US) (Pierson et al., 1999) and a saline lake from Camargue, France (Wieland et al., 2005) using the reported pH, O_2_ and Fe^2+^ concentrations. The calculated half-life of Fe^+2^ at the surface of the mats described by Pierson et al. (1999) ranges between 19 and 35 ms. Since the data provided by these authors do not include pH and O_2_ profiles in the water column above the mats, full iron profiles could not be reconstructed. Interestingly, the same study reported stimulation of O_2_ production by an increasing concentration of Fe^2+^, similar to what we have found for the cyanobacteria from our non-aerated Fe^2+^-rich reactor. With respect to the Camargue saline lake, Fe^2+^ diffuses toward the cyanobacterial populations at the surface of the mats from deeper sediment layers (Wieland et al., 2005). The lake water is oxic and has a pH >8.9, thus Fe^2+^ would not be stable under these conditions (Morgan and Lahav, 2007). Nevertheless, calculated half-life of Fe^2+^ in these mats ranges between 2 and 8 ms. In both cases the short (calculated) half-life of Fe^2+^ in the microenvironment of the cyanobacteria suggests that they are not exposed to high concentration of Fe^2+^.

We propose that cyanobacteria living in benthic communities in Fe^2+^-rich environments require high photosynthesis rates to avoid self-encrustation in iron precipitates. This attribute combined with mass transfer resistance will lead to elevated pH and O_2_ concentrations, which will form a barrier that prevents Fe from precipitating directly on the cells. Coupled with the flow conditions of natural water bodies the majority of the abiotically precipitated iron will be dispersed by the water. The high rate of photosynthesis will ensure that the barrier will be established within seconds to minutes in case flow conditions or iron concentrations in the ambient water are fluctuating.

Under the experimental conditions in the Äspö HRL, the barrier is present continuously because of the uninterrupted exposure to light, which would not be the case under natural conditions due to diurnal light fluctuations. Nevertheless, such light fluctuations should not have a significant effect on the ability of cyanobacteria to prevent self-encrustation in iron. Fe^2+^-rich environments, such as the one in the Äspö HRL, typically have very low O_2_ concentrations (Pierson et al., 1999; Ionescu et al., 2014a). Thus, in the dark, when there is no photosynthetic activity, the microenvironment in the cyanobacterial biofilms will rapidly turn anoxic due to the combined effects of respiration and mass transfer resistance, and the cells will not face an encrustation problem. The need for a protection mechanism against iron encrustation returns as soon as the photosynthetic activity resumes upon onset of illumination. Here, a high rate of photosynthesis to minimize the exposure time to high Fe^2+^ while producing O_2_ will be essential.

In addition to iron self-encrustation, cyanobacteria from Fe^2+^-rich environments need to deal also with Fe^2+^ toxicity (Brown et al., 2005; Shcolnick and Keren, 2006). While the iron-barrier provides a protection against iron toxicity during day time, it will have no effect in the dark. Therefore, we suggest that these cyanobacteria require a second mechanism to deal with the high Fe^2+^ concentrations at night, such as elaborate metal export systems (Shcolnick et al., 2009).

Single cells or biofilms below a critical biomass appear to get encrusted in iron (Pierson et al., 1999). The amount of biomass necessary to create the suggested “iron barrier” probably cannot be reached under the conditions in which the cells would need such a barrier. Hence, we suggest that new biofilms of the required critical biomass are formed in a “Fe^2+^ neutral” environment (e.g., floating on the surface or on the shores of a water body). When such biofilms become permanently exposed to high Fe^2+^, their survival is determined by the rapid achievement of a steady state in the distribution of Fe^2+^ in the microenvironment around the biofilms. This in turn depends on biomass, the overall photosynthesis rate of the biofilms and the ability of the biofilms to respond to the change in Fe^2+^ concentrations. This also suggests that a biofilm that contains a critical biomass of “iron tolerant cyanobacteria” may shelter less tolerant species, which could explain the observed partial overlap between the cyanobacterial communities from the aerated and non-aerated Fe^2+^-rich reactors.

The ability of cyanobacteria to survive in Fe^2+^-rich environments without getting encrusted while at the same time facilitating precipitation of iron oxides through their O_2_ producing photosynthetic activity may have implications for our understanding of early Earth geology. Cyanobacteria have been often suggested to be involved in the creation of the banded iron formations (BIF), the largest Fe depositions on Earth. Whether their impact was directly in the water body (Cloud, 1965, 1973) or indirectly by oxygenation of the atmosphere remains controversial (Cloud, 1968). However, despite cyanobacterial microfossils being found in BIFs (Cloud, 1965), the overall abundance of organic matter is scarce (Bontognali et al., 2013). If cyanobacteria were directly involved in the formation of BIFs, the existence of a cyanobacterial protective “iron barrier” may explain the lack of biomass in the Fe precipitates. Such a barrier mechanism would prevent the cyanobacteria from becoming entrapped in the precipitated iron. The Fe^2+^ supplied from the deep primordial ocean (Wang et al., 2009) would have been oxidized in the water column below the chlorophyll maximum and sink down as particulate Fe(III) without the carryover of biomass. Intuitively, marine cyanobacteria appear to be suitable model organisms to study the existence of such a mechanism. Nevertheless, modern oceans have been poor in iron for about 2 Ga, a substantial period for evolutionary adaptation, which makes it unlikely for marine cyanobacteria today to be adapted to such elevated Fe^2+^ concentrations. Hence, in this case, cyanobacteria from brackish-saline, iron-rich environments such as the Äspö HRL still provide the best model organisms for studying ancient Earth marine analogs.

### Conflict of interest statement

The authors declare that the research was conducted in the absence of any commercial or financial relationships that could be construed as a potential conflict of interest.

## Supplementary material

Figure S1 **Pictures of an aerated (A) and a non-aerated (B) reactor set up in the ÄSPÖ Hard Rock Laboratory**. The draining tube of the non-aerated reactor was bended such that flow-through was obtained only when the reactor was full to the top.

Figure S2 **(A)** Experimental setup for laboratory microsensor measurements. N_2_-purged medium was run through a small flow chamber (V~5 ml) **(B)** in which filters overgrown with cyanobacterial biofilms were placed and measured using microsensors **(C)**.

Figure S3 **Maximum Likelihood tree of cyanobacterial sequences from the different reactors together with reference sequences**. The sequences are labeled according to their origin: 1327 R1 and R2 for the Fe^2+^-rich aerated and non-aerated reactors, respectively; TASF R1 for the Fe^2+^-poor aerated reactor. Bold labels were used in the case of larger clusters of sequences of measured cultures.

Figure S4 **O_2_ microprofiles measured in the cyanobacterial biofilms in the aerated and non-aerated Fe^2+^-rich reactors in the dark (filled symbols) and at incident irradiance of 60 μmol photons m^−2^ s^−1^ (open symbols)**. The bars represent gross photosynthesis rates (in μM O_2_ s^−1^) as measured by the light-dark-shift method in 100 μm steps in the biofilm. All measurements were done *in-situ*.

Figure S5 ***In-situ* O_2_ and pH microprofiles measured in the cyanobacterial biofilm from the aerated Fe^2+^-poor reactor before and after the addition of 25 μM of Fe^2+^ at time points indicated in the legend**. All profiles were measured using similar light intensity as used in the reactors. The measurements were conducted outside of the reactor using the natural water. N_2_ gas was bubbled continuously to maintain anoxic conditions.

Figure S6 **Nanoscale Secondary Ion Mass Spectrometry (nanoSIMS) analysis of a cyanobacterial enrichment culture and an iron oxidizing mat obtained from the non-aerated Fe^2+^-rich reactor and incubated with ^57^Fe^2+^**. The Secondary electron panels **(A,C)** show the surface topography with color bar representing signal intensity. No enrichment with ^57^Fe^2+^ can be seen near or on the cyanobacterial filaments **(B)**, while an overall high concentration of ^57^Fe^2+^ was detected in the iron oxidizing mat including a highly enriched single filament **(D)**.
