# Supplementary figures and images for: Oxygenic photosynthesis as a protection mechanism for cyanobacteria against iron-encrustation in environments with high Fe2+ concentrations
Source: Front Microbiol. 2014 Sep 2;5:459. doi: 10.3389/fmicb.2014.00459 (PMC4151041; doi:10.3389/fmicb.2014.00459)

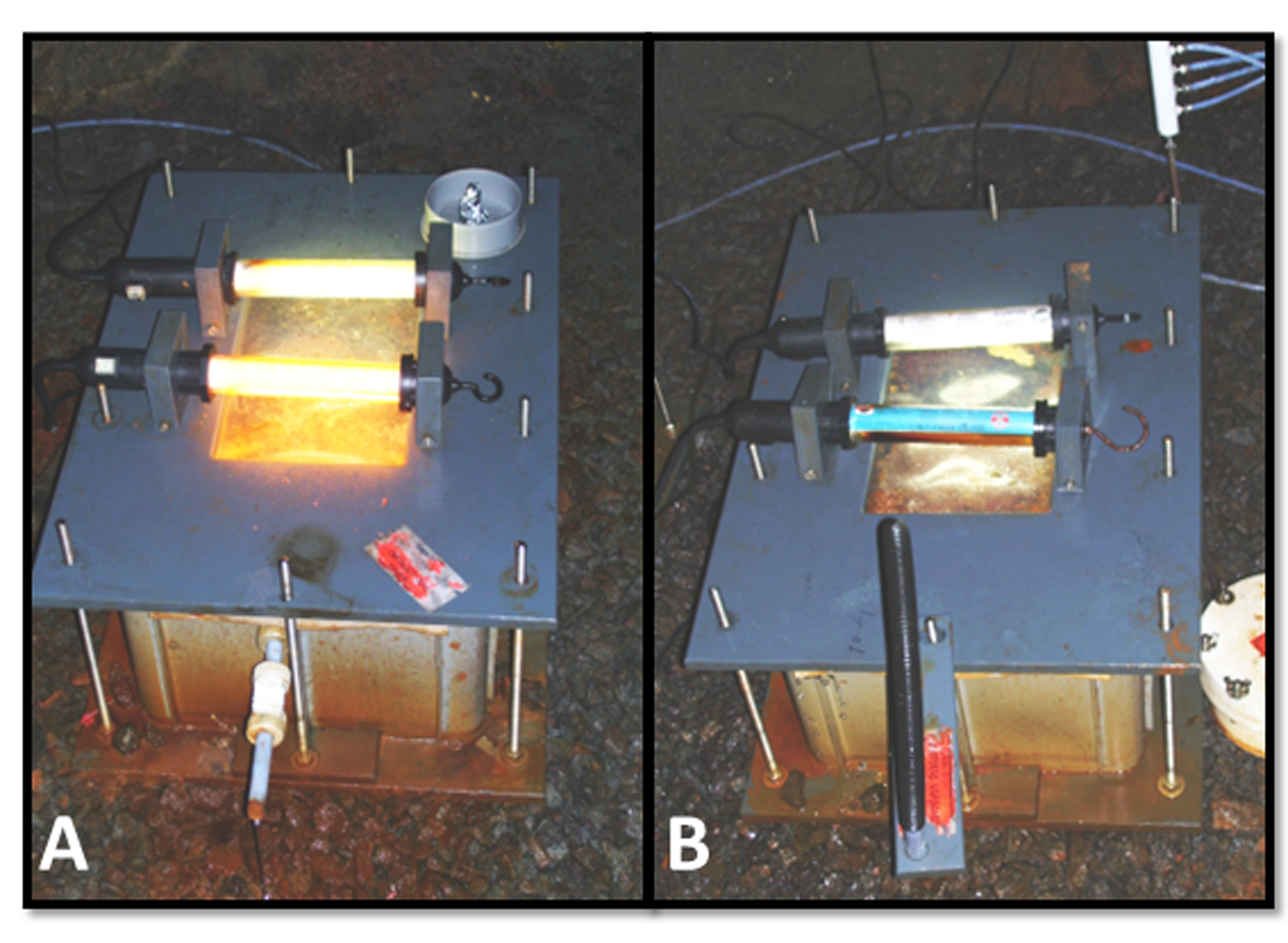

Supplement: Figure S1 — Pictures of an aerated (A) and a non-aerated (B) reactor set up in the ÄSPÖ Hard Rock Laboratory. The draining tube of the non-aerated reactor was bended such that flow-through was obtained only when the reactor was full to the top. [file Image1.TIF]

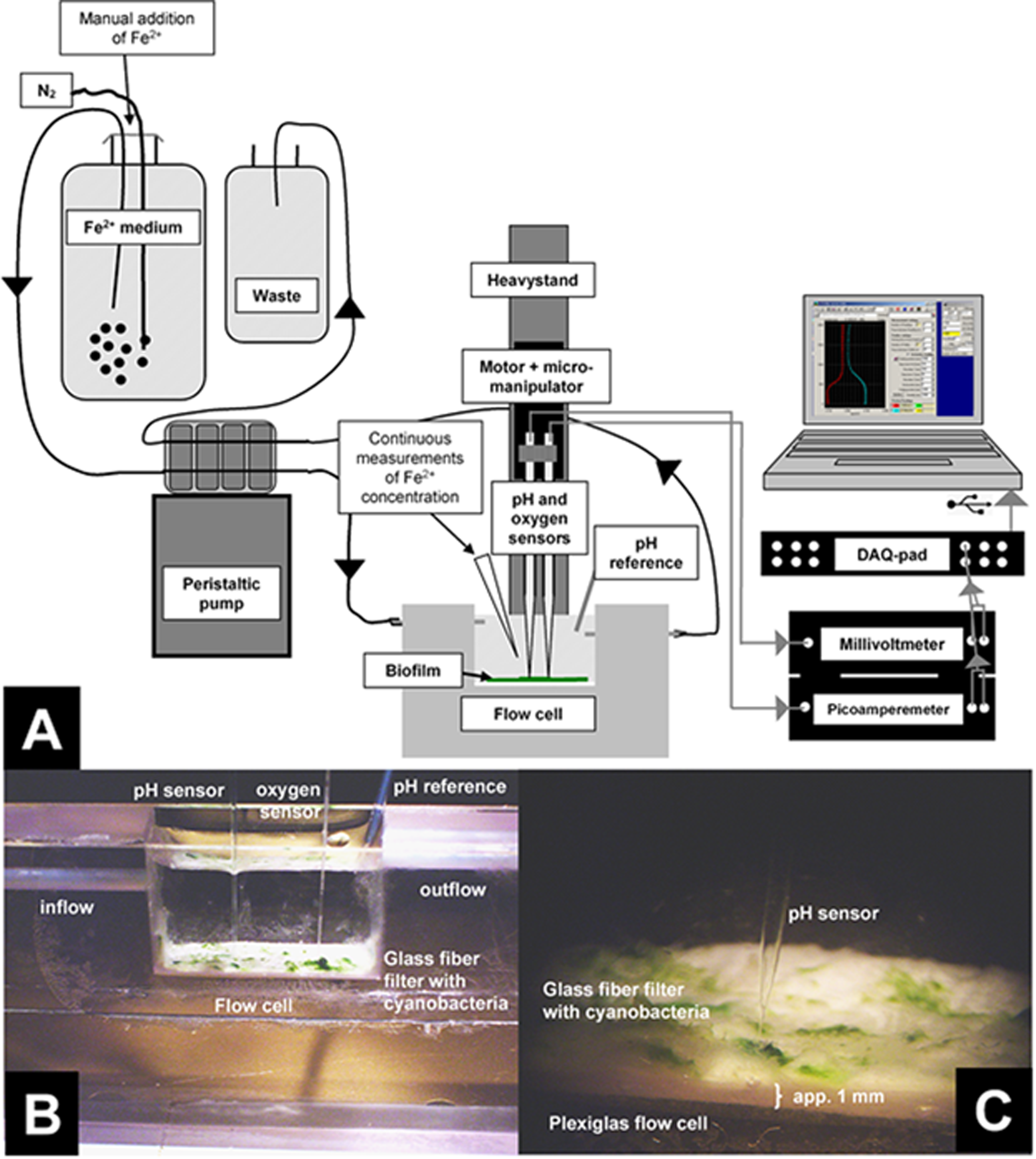

Supplement: Figure S2 — (A) Experimental setup for laboratory microsensor measurements. N2-purged medium was run through a small flow chamber (V~5 ml) (B) in which filters overgrown with cyanobacterial biofilms were placed and measured using microsensors (C). [file Image2.TIF]

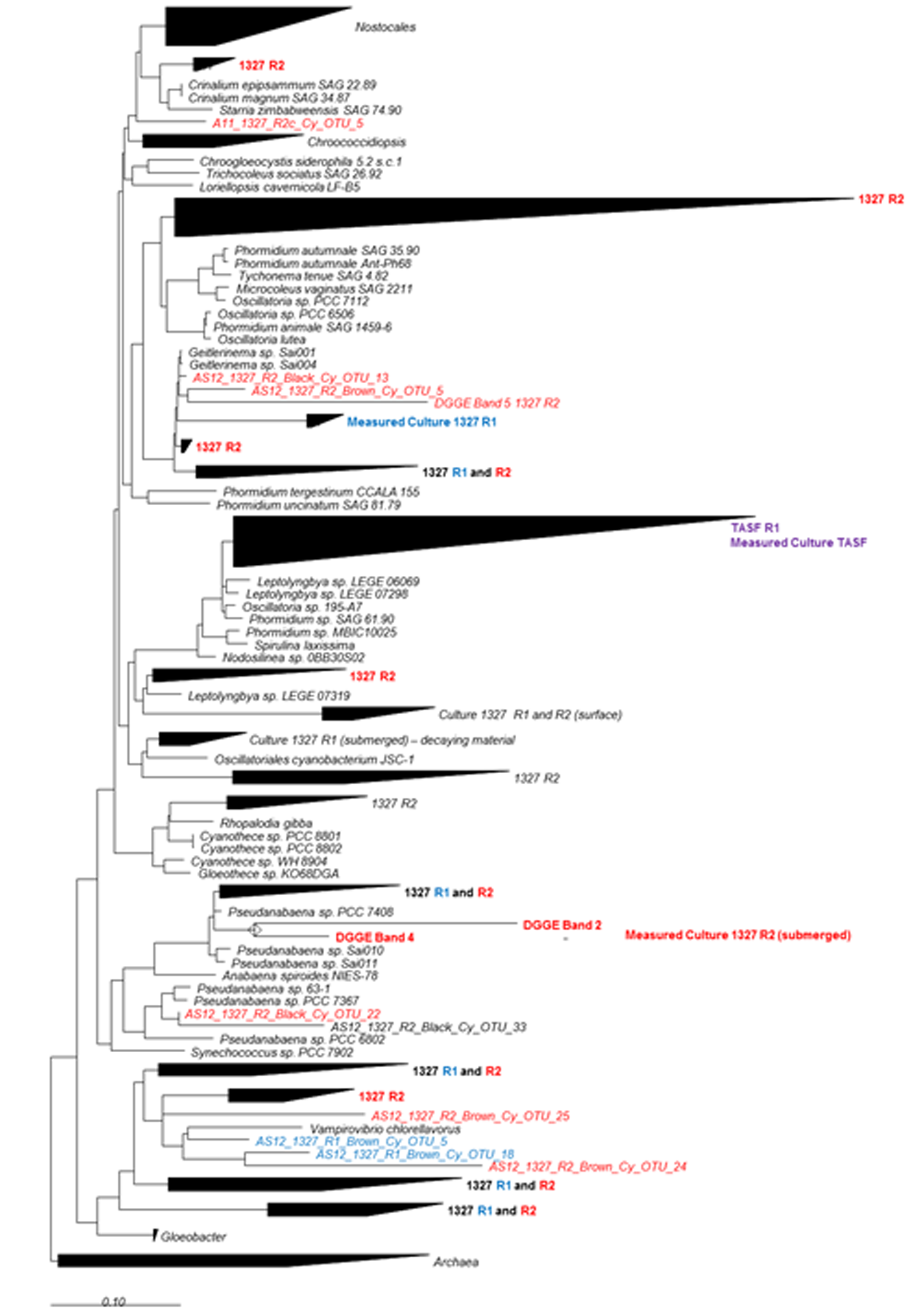

Supplement: Figure S3 — Maximum Likelihood tree of cyanobacterial sequences from the different reactors together with reference sequences. The sequences are labeled according to their origin: 1327 R1 and R2 for the Fe2+-rich aerated and non-aerated reactors, respectively; TASF R1 for the Fe2+-poor aerated reactor. Bold labels were used in the case of larger clusters of sequences of measured cultures. [file Image3.TIF]

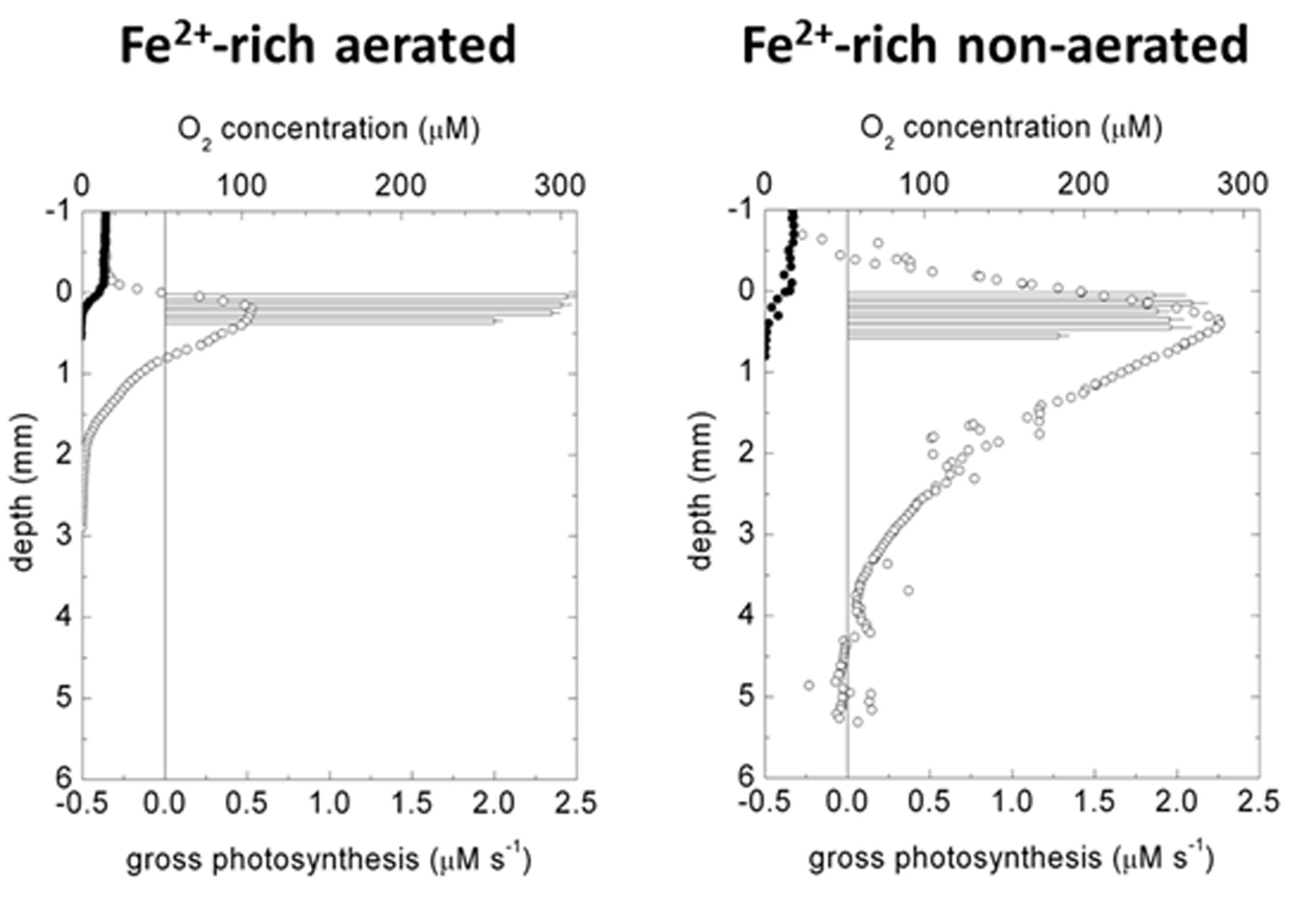

Supplement: Figure S4 — O2 microprofiles measured in the cyanobacterial biofilms in the aerated and non-aerated Fe2+-rich reactors in the dark (filled symbols) and at incident irradiance of 60 μmol photons m−2 s−1 (open symbols). The bars represent gross photosynthesis rates (in μM O2 s−1) as measured by the light-dark-shift method in 100 μm steps in the biofilm. All measurements were done in-situ. [file Image4.TIF]

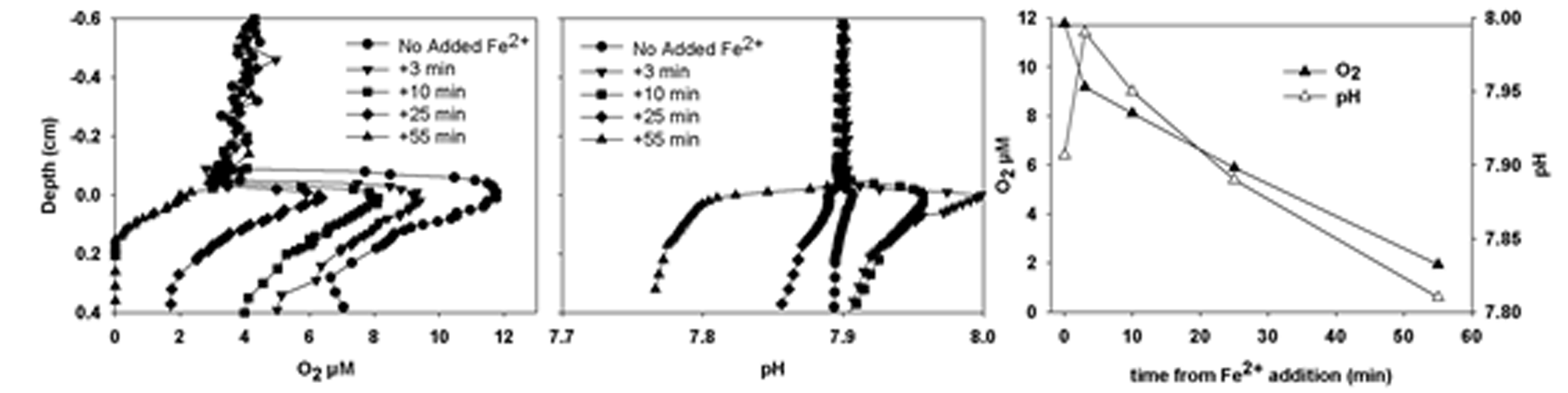

Supplement: Figure S5 — In-situ O2 and pH microprofiles measured in the cyanobacterial biofilm from the aerated Fe2+-poor reactor before and after the addition of 25 μM of Fe2+ at time points indicated in the legend. All profiles were measured using similar light intensity as used in the reactors. The measurements were conducted outside of the reactor using the natural water. N2 gas was bubbled continuously to maintain anoxic conditions. [file Image5.TIF]

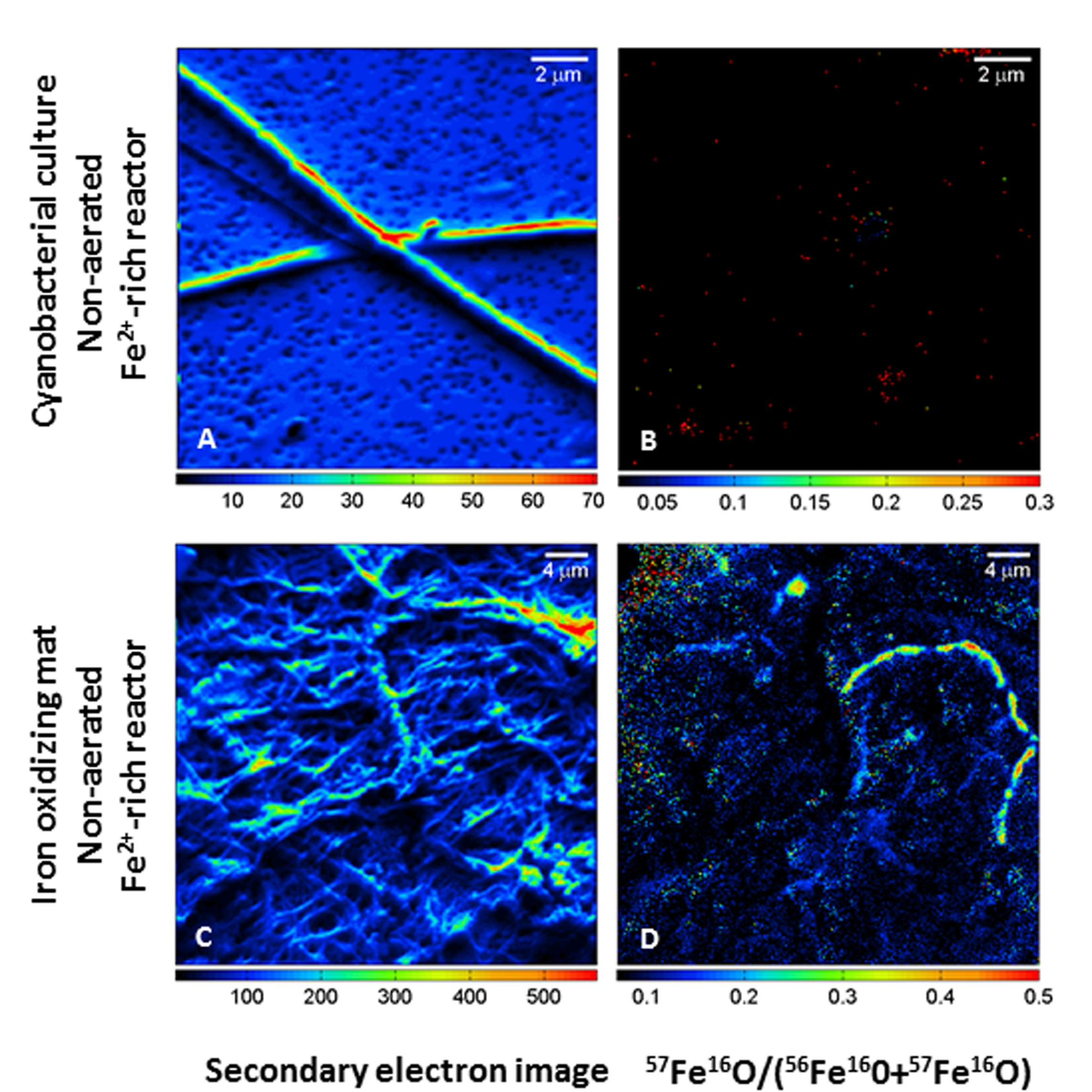

Supplement: Figure S6 — Nanoscale Secondary Ion Mass Spectrometry (nanoSIMS) analysis of a cyanobacterial enrichment culture and an iron oxidizing mat obtained from the non-aerated Fe2+-rich reactor and incubated with 57Fe2+. The Secondary electron panels (A,C) show the surface topography with color bar representing signal intensity. No enrichment with 57Fe2+ can be seen near or on the cyanobacterial filaments (B), while an overall high concentration of 57Fe2+ was detected in the iron oxidizing mat including a highly enriched single filament (D). [file Image6.TIF]
